# Supplementary material for: Correlation-driven nonequilibrium exciton site transition in a WSe2/WS2 moiré supercell
Source: Nat Commun. 2024 Apr 17;15:3312. doi: 10.1038/s41467-024-47768-6 (PMC11024152; doi:10.1038/s41467-024-47768-6)
Supplement: Supplementary file 1 — Supplementary information [file 41467_2024_47768_MOESM1_ESM.pdf]

Supplementary Information for

Correlation-driven nonequilibrium exciton site transition in a WSe_2/WS_2 moiré supercell

Jinjae Kim[1,2,†], Jiwon Park[1,2,†], Hyojin Choi[1,2], Taeho Kim[3,4], Soonyoung Cha[5], Yewon Lee[3,4], Kenji Watanabe[6], Takashi Taniguchi[6], Jonghwan Kim[3,4], Moon-Ho Jo[3,4], and Hyunyong Choi[1,2,*]

[1]Department of Physics, Seoul National University, Seoul 08826, Korea

[2]Institute of Applied Physics, Seoul National University, Seoul 08826, Korea

[3]Department of Materials Science and Engineering, Pohang University of Science and Technology, Pohang 37673, Korea.

[4]Center for van der Waals Quantum Solids, Institute for Basic Science (IBS), Pohang 37673, Republic of Korea

[5]Department of Physics and Astronomy, University of California, Riverside, California 92521, United States.

[6]Advanced Materials Laboratory, National Institute for Materials Science, 1-1 Namiki, Tsukuba 305-0044, Japan

[†]These authors contributed equally to this work.

[*]Corresponding author: hy.choi@snu.ac.kr

Content

Supplementary Notes

1. Spectral blueshift of the intralayer exciton X_1, X_2, and X_3
2. Comparison of the electron-exciton Coulomb repulsion ($U_{\mathrm{e-ex}}$) and the energy gap (ΔE_{g}) between two moiré local minima
3. Estimation of exciton density by using a transfer-matrix calculation
4. A fit function of population dynamics with a finite rise time and biexponential decay

Supplementary Tables

Supplementary Figures

Supplementary Notes

1. Spectral blueshift of the intralayer exciton X_1, X_2, and X_3

The WSe_2/WS_2 hetorobilayer exhibits a type II band alignment, where electrons are located in the WS_2 layer, while holes are in the WSe_2 layer. The charge transfer into different layers is known to be very efficiency, occurring within a few hundred femtoseconds[1-3]. Because we use the probe photon energy near the WSe_2 A exciton energy, our investigations focus on the behavior of holes[4] in the WSe_2 layer (see Supplementary Fig. 14). Therefore, understanding how the hole wavefunctions are distributed in the moiré supercell is crucial. According to the recent calculations[5] in the R-stacked WSe_2/WS_2 moiré superlattices, the hole charges of the intralayer excitons X_1, X_2, and X_3 (shown in Fig. 2a in the main text) reside predominantly at the $\mathrm{R_h^h}$, $\mathrm{R_h^X}$, and $\mathrm{R_h^X}$ site, respectively.

In the R-stacking, the interlayer holes are vertically aligned[6] with interlayer electrons at the $\mathrm{R_h^X}$ site. The photo-generated interlayer holes by the pump pulses also occupy the $\mathrm{R_h^X}$ site in the WSe_2 layer. Consequently, the holes of the intralayer exciton X_1 occupy a different site, i.e. $\mathrm{R_h^h}$ site, from the interlayer exciton site, i.e. $\mathrm{R_h^X}$ site, in the neutral regime. However, in the n-doping regime, the interlayer excitons make a spatial transition to the $\mathrm{R_h^h}$ site, accompanied with polarization switching, as described in the main text. Thus, it results in a pronounced blueshift near the X_1 resonance because the interlayer excitons and the hole of intralayer exciton X_1 occupy the same $\mathrm{R_h^h}$ site in the n-doping regime.

In the charge neutral regime, because the holes of the intralayer excitons X_2 and X_3 occupy primarily at $\mathrm{R_h^X}$ site (the same site as interlayer excitons in the neutral regime), it leads to a spectral

blueshift near the X_2 and X_3 resonances. In the n-doping regime, due to the exciton site transition to the $\mathrm{R_h^h}$ site, the spectral blueshift becomes obscure near the X_2 and X_3 resonances in contrast with the X_1 resonance (see Fig. 4d of the main text).

2. Comparison of the electron-exciton Coulomb repulsion ($U_{\mathrm{e-ex}}$) and the energy gap (ΔE_{g}) between two moiré local minima

In this section, we qualitatively estimate the energy gap (ΔE_{g}) between two local minima and the electron-exciton Coulomb repulsion ($U_{\mathrm{e-ex}}$) in a moiré supercell. First, the exciton site transition driven by strong correlations is possible if $U_{\mathrm{e-ex}} > \Delta E_{\mathrm{g}}$. Because $U_{\mathrm{e-ex}}$ is dominated by a short-range Coulomb interaction, the interactions between particles within the same layer will be significantly stronger than those involving particles from different layers. As a result, we expect that the energy scale of $U_{\mathrm{e-ex}}$ and the electron-electron Coulomb repulsion ($U_{\mathrm{e-e}}$) is to be on the same order. Meanwhile, experimental results and theoretical predictions for $U_{\mathrm{e-e}}$ are typically within a range of 20~100 meV[7-9]. A simple estimation of $U_{\mathrm{e-e}}$ can be derived from $\frac{e^2}{\epsilon a_{\mathrm{M}}}$, from which one can obtain approximately 45 meV[8,10]. Here, ϵ (~4) is the dielectric constant of hBN and a_{M} (~8 nm) is the moiré periodicity; the size of wavefunction localization is approximately a_{M}. Furthermore, we estimate $U_{\mathrm{e-ex}}$ from the abrupt blueshift of PL at $\nu = \pm 1$ (which is induced by $U_{\mathrm{e-ex}}$). The measurements yield within the range of 30~50 meV[6,10] for $U_{\mathrm{e-ex}}$, which is comparable to $U_{\mathrm{e-e}}$.

Meanwhile, the insulating gap at $\nu = 1$ in WSe_2/WS_2, as inferred from the thermal activation[9,11], appears to be relatively small, typically of around 10 meV. This value is smaller than the estimated Coulomb repulsion $U_{\mathrm{e-ex}}$. Notably, a prior theoretical study[12] suggests that this thermally activated gap is comparable to the charge-transfer gap (ΔE_{g}), indicating that the system

is indeed charge transfer insulating states when $\nu > 1$. As a result, the condition $U_{\mathrm{e-ex}} > \Delta E_{\mathrm{g}}$ holds, leading to a correlation-driven exciton site transition.

3. Estimation of exciton density using a transfer-matrix calculation

To estimate the photoexcited exciton density, we employ the general transfer-matrix method technique[13,14]. The amplitude reflection coefficient of the sample is given by the form of the summation of independent Lorentz oscillators[15]

$$r(\hbar\omega) = \sum_i \frac{i\Gamma_{\mathrm{rad},i}}{E_i - \hbar\omega - i(\Gamma_{\mathrm{rad},i} + \Gamma_{\mathrm{nonrad},i})},$$

where E_i is the resonance energy, $\Gamma_{\mathrm{rad},i}$ and $\Gamma_{\mathrm{nonrad},i}$ are the radiative and nonradiative dampings of the i-th exciton. We have adjusted these three parameters until the calculated reflection contrast spectrum matches the experimental data. The fit results are shown in Supplementary Fig. 17, and the obtained fitting parameters are summarized in Supplementary Table 3.

Because of the spectral mismatch between the absorption and the pump (see Supplementary Fig. 18), we have conducted additional calculations to estimate the total absorption (A_{tot})[16]. A_{tot} is the weighted average absorption with the pump spectrum, i.e.

$$A_{\mathrm{tot}} = \frac{\int I_{\mathrm{pump}}(E) A(E) dE}{\int I_{\mathrm{pump}}(E) dE} = 0.0748,$$

where $I_{\mathrm{pump}}(E)$ is the pump spectrum and $A(E)$ is the absorption spectrum of the sample extracted from the transfer-matrix calculation. The measured pump fluence F_{pump} is converted to the incident photon density n_{pump}, and then converted to the pump-induced exciton density $n_{\mathrm{ex}} = A_{\mathrm{tot}} n_{\mathrm{pump}}$. By using this procedure, we estimate $n_{\mathrm{ex}} = 2.79 \times 10^{12}\ \mathrm{cm}^{-2}$ for $F_{\mathrm{pump}} = 12\ \mu\mathrm{J}/$

cm^2. Corresponding exciton density is comparable with the moiré density, implying the one-to-one correspondence between excitons and moiré unit cells. For this case, there is roughly one exciton for every moiré unit cell.

4. A fit function of population dynamics with a finite rise time and biexponential decay

Here we follow the notation of reference 17. We assume that the observed dynamics can be effectively modeled by a biexponential decay (time constants τ_1 and τ_2) and a finite rise time (τ_R). This model is commonly used for describing thermalization and recombination dynamics in various semiconductor structures. Expressed in the time domain, the signal $\mathrm{g}_0(t)$ takes the form of

$$\mathrm{g}_0(t) = \Theta(t)\left[1 - \exp\left(-\frac{t}{\tau_\mathrm{R}}\right)\right]\left[A_1 \exp\left(-\frac{t}{\tau_1}\right) + A_2 \exp\left(-\frac{t}{\tau_2}\right)\right], \qquad \text{(S1)}$$

where A_1 and A_2 are the amplitudes of the two temporal components, and the prefactor $\Theta(t)$ is the Heaviside step function. For the pump pulse profile $\mathrm{p}_\mathrm{pump}(t)$, the temporal profile of a typical mode-locked laser pulse is characterized by a Gaussian function, given by

$$\mathrm{p}_\mathrm{pump}(t) = B_\mathrm{pu} \exp\left(-\frac{t^2}{\sigma_\mathrm{pu}^2}\right) \sin\left(2\pi\nu_\mathrm{pu} t\right), \qquad \text{(S2)}$$

where σ_pu is a pulse width, ν_pu is a central frequency, and B_pu is an amplitude. Mathematically, the actual material dynamics involve a convolution integral between $\mathrm{g}_0(t)$ and $\mathrm{p}_\mathrm{pump}(t)$, which can be represented by

$$\mathrm{g}_1(t) = \int_{-\infty}^{\infty} dY\, \mathrm{g}_0(Y)\mathrm{p}_\mathrm{pump}(Y - t). \qquad \text{(S3)}$$

Because the finite width of the probe pulse also causes a modification in the detected signal, we conduct the same convolution procedure to $\mathrm{g}_1(t)$. Similar to $\mathrm{p}_\mathrm{pump}(t)$, the probe pulse profile $\mathrm{p}_\mathrm{probe}(t)$ can be defined in the form of

$$\mathrm{p_{probe}}(t) = B_{\mathrm{pr}} \exp\left(-\frac{t^2}{\sigma_{\mathrm{pr}}^2}\right) \sin(2\pi\nu_{\mathrm{pr}} t), \tag{S4}$$

where σ_{pr} is a pulse width, ν_{pr} is a central frequency, and B_{pr} is an amplitude of probe pulse. Next, a second convolution integral of $\mathrm{g_1}(t)$ and $\mathrm{p_{probe}}(t)$ can be obtained by

$$\begin{aligned}
\mathrm{g_2}(t) &= \int_{-\infty}^{\infty} dX\, \mathrm{g_1}(X)\mathrm{p_{probe}}(X - t) \\
&= \int_{-\infty}^{\infty} dX \left[\int_{-\infty}^{\infty} dY\, \mathrm{g_0}(Y)\mathrm{p_{pump}}(Y - X)\right] \mathrm{p_{probe}}(X - t) \\
&= \int_{-\infty}^{\infty} dY\, \mathrm{g_0}(Y) \left[\int_{-\infty}^{\infty} dX\, \mathrm{p_{pump}}(Y - X)\mathrm{p_{probe}}(X - t)\right].
\end{aligned} \tag{S5}$$

In equation (S5), the order of integration can be exchanged, yielding a cross-correlation integral, $C(t)$, which can be shortened by

$$\begin{aligned}
C(t) &= \int_{-\infty}^{\infty} dZ\, \mathrm{p_{pump}}(Z)\mathrm{p_{probe}}(Z - t) \\
&= \int_{-\infty}^{\infty} dZ\, B_{\mathrm{pu}} \exp\left(-\frac{Z^2}{\sigma_{\mathrm{pu}}^2}\right) B_{\mathrm{pr}} \exp\left(-\frac{(Z - t)^2}{\sigma_{\mathrm{pr}}^2}\right) \\
&= B_{\mathrm{pu}} B_{\mathrm{pr}} \exp\left(-\frac{t^2}{2\sigma^2}\right) \\
&\equiv C_0 \exp\left(-\frac{t^2}{w^2}\right),
\end{aligned} \tag{S6}$$

where we assume $\sigma_{\mathrm{pu}} \cong \sigma_{\mathrm{pr}} \equiv \sigma$, $w = \sigma\sqrt{2}$ and $C_0 = B_{\mathrm{pu}} B_{\mathrm{pr}}$. Using this result, the actual signal $\mathrm{g_2}(t)$ can be written in the form of

$$
\begin{aligned}
g_2(t) &= \int_{-\infty}^{\infty} dY\, g_0(Y) C(Y-t) \\
&= C_0 \int_0^{\infty} dY\, \left[1 - \exp\left(-\frac{Y}{\tau_R}\right)\right]\left[A_1 \exp\left(-\frac{Y}{\tau_1}\right)\right. \\
&\qquad \left. + A_2 \exp\left(-\frac{Y}{\tau_2}\right)\right] \exp\left(-\frac{(Y-t)^2}{w^2}\right).
\end{aligned}
\tag{S7}
$$

This integral can be simplified using the error function integral $\mathrm{erf}(x)$, resulting in

$$
\begin{aligned}
g_2(t) = {} & D_1 \exp\left(-\frac{t}{\tau_1}\right)\left[1 - \mathrm{erf}\left(\frac{w}{2\tau_1} - \frac{t}{w}\right)\right] \\
& + D_2 \exp\left(-\frac{t}{\tau_2}\right)\left[1 - \mathrm{erf}\left(\frac{w}{2\tau_2} - \frac{t}{w}\right)\right] \\
& - D_1 \exp\left[-t\left(\frac{1}{\tau_1} + \frac{1}{\tau_R}\right)\right]\left[1 - \mathrm{erf}\left[\frac{w}{2}\left(\frac{1}{\tau_1} + \frac{1}{\tau_R}\right) - \frac{t}{w}\right]\right] \\
& - D_2 \exp\left[-t\left(\frac{1}{\tau_2} + \frac{1}{\tau_R}\right)\right]\left[1 - \mathrm{erf}\left[\frac{w}{2}\left(\frac{1}{\tau_2} + \frac{1}{\tau_R}\right) - \frac{t}{w}\right]\right],
\end{aligned}
\tag{S8}
$$

where $D_1 = C_0 A_1$ and $D_2 = C_0 A_2$.

	R_h^h	R_h^X	R_h^M
$\|K, v_\uparrow\rangle \rightarrow \|K, c'_\downarrow\rangle$	z	σ^+	σ^-
$\|K, v_\uparrow\rangle \rightarrow \|K, c'_\uparrow\rangle$	σ^+	σ^-	z
$\|-K, v_\downarrow\rangle \rightarrow \|-K, c'_\uparrow\rangle$	z	σ^-	σ^+
$\|-K, v_\downarrow\rangle \rightarrow \|-K, c'_\downarrow\rangle$	σ^-	σ^+	z

Supplementary Table 1 | Optical selection rules of singlet- and triplet-interlayer excitons for R-stacking. Optical selection rules of interlayer excitons not only depend on the spin-valley indices but also local atomic registry in R-stacking[6,18-21], where K ($-$K) is the $\pm$K valley index, c′ (v) is the conduction (valence) band of WS_2 (WSe_2) and the vertical arrows (↑ and ↓) are the spin index. The schematic for each band is illustrated in Supplementary Fig. 15.

	H_h^h	H_h^X	H_h^M
$\|K, v_\uparrow\rangle \rightarrow \|-K, c'_\uparrow\rangle$	σ^-	σ^+	z
$\|K, v_\uparrow\rangle \rightarrow \|-K, c'_\downarrow\rangle$	σ^+	z	σ^-
$\|-K, v_\downarrow\rangle \rightarrow \|K, c'_\downarrow\rangle$	σ^+	σ^-	z
$\|-K, v_\downarrow\rangle \rightarrow \|K, c'_\uparrow\rangle$	σ^-	z	σ^+

Supplementary Table 2 | Optical selection rules of singlet- and triplet-interlayer excitons for H-stacking. Same table as Supplementary Table 1, but for the H-stacking[6,18-21]. The schematic for each band is illustrated in Supplementary Fig. 16.

	E (eV)	Γ_{rad} (meV)	Γ_{nonrad} (meV)
Oscillator 1	1.685	0.072	3
Oscillator 2	1.745	0.03	10
Oscillator 3	1.79	0.05	10
Oscillator 4	1.868	0.04	20
Oscillator 5	2.013	0.4	15
Oscillator 6	2.145	1.2	70

Supplementary Table 3 | The fitting parameters determined by the transfer-matrix method. During the fitting process, we adopt the Lorentz oscillator model for each exciton, which presumes that the exciton resonances are well separated. The oscillators 1, 2, and 3 correspond to moiré intralayer excitons near the WSe_2 A_{1s} exciton resonance (~1.7 eV). The oscillators 4 and 6 correspond to the A_{2s} exciton and B_{1s} exciton of WSe_2, respectively. The oscillator 5 corresponds to the A_{1s} exciton of WS_2 near the energy of 2.0 eV.

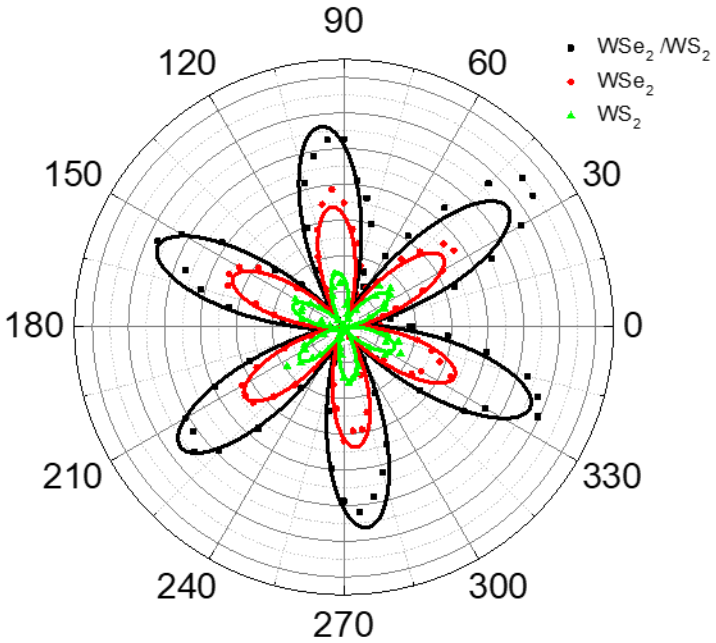

Supplementary Figure 1 | Determination of crystal orientation by angle-resolved second harmonic generation spectroscopy. Solid red circles, green triangles, and black rectangles are the polarization-dependent SHG data from monolayer WSe_2, WS_2, and WSe_2/WS_2 heterobilayer regions, respectively. The solid curve is a fit to the data using $y = y_0 + A\sin^2\left[\frac{\pi}{w}(x - x_c)\right]$, where x is the excitation polarization angle and x_c is the crystal orientation. While w is fixed with 60°, y_0 and A are the free fitting parameters. The twist angle between WSe_2 and WS_2 is determined to be $1.2° \pm 0.6°$ for the device R1. When the two monolayers are R-stacked (nearly 0°), SHG from the heterobilayer is much stronger than the monolayer.

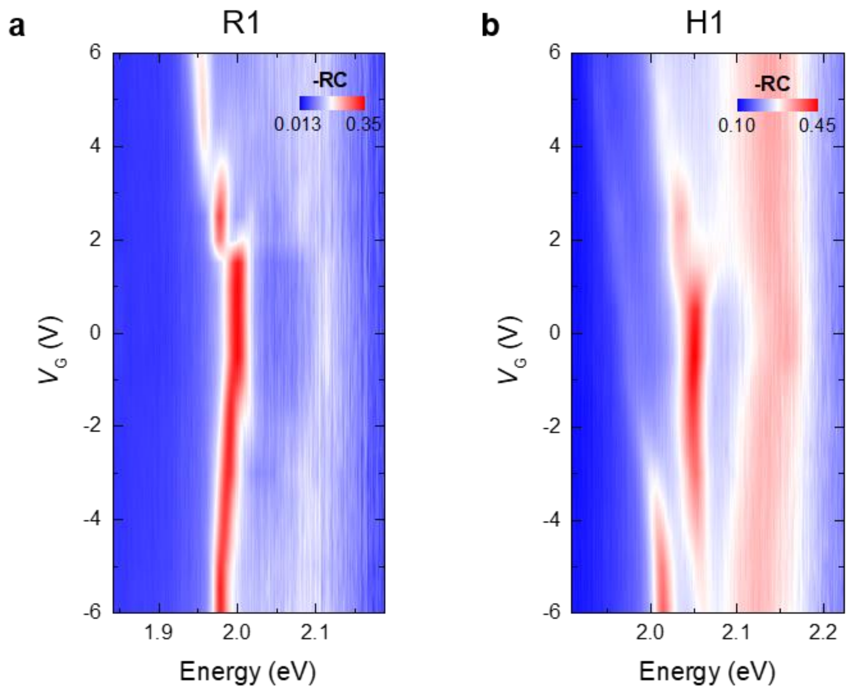

Supplementary Figure 2 | Gate-dependent reflection contrast on R1 and H1 device. a RC data on the R1 device with the probe photon energy close to the A exciton resonance of WS_2. **b** The same RC measurement on the device H1. Energy level anti-crossing and redistribution of oscillator strength between interlayer exciton and intralayer exciton are observed, indicating the formation of the hybridized excitons in the H-stacked WSe_2/WS_2 heterobilayers[12].

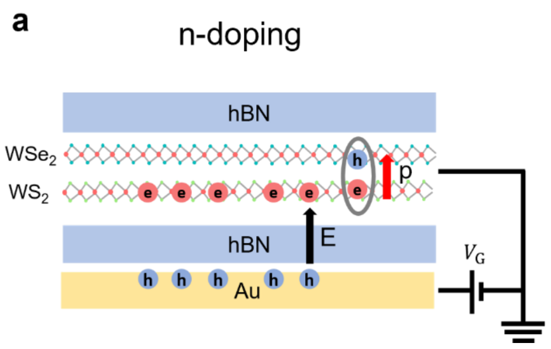

Supplementary Figure 3 | Schematics of the back-gated WSe_2/WS_2 heterostructure device. The WSe_2/WS_2 heterostructure exhibits a type II band alignment; electrons are preferentially in the WS_2 layer and holes in the WSe_2 layer. For the n-doping case **a** electrons are accumulated within the WS_2 layer, which effectively screen the applied electric field. Conversely, when V_G is reversed to the p-doping **b** holes are accumulated in the WSe_2 layer. Given that the WS_2 layer is intrinsically undoped, its contribution to electric field screening is minimal. Consequently, the electric field between the WSe_2 and WS_2 layers becomes greater than that in the n-doping region. Consequently, the Stark shift-induced blueshift is significant in the p-doping regime, but small in the n-doping regime.

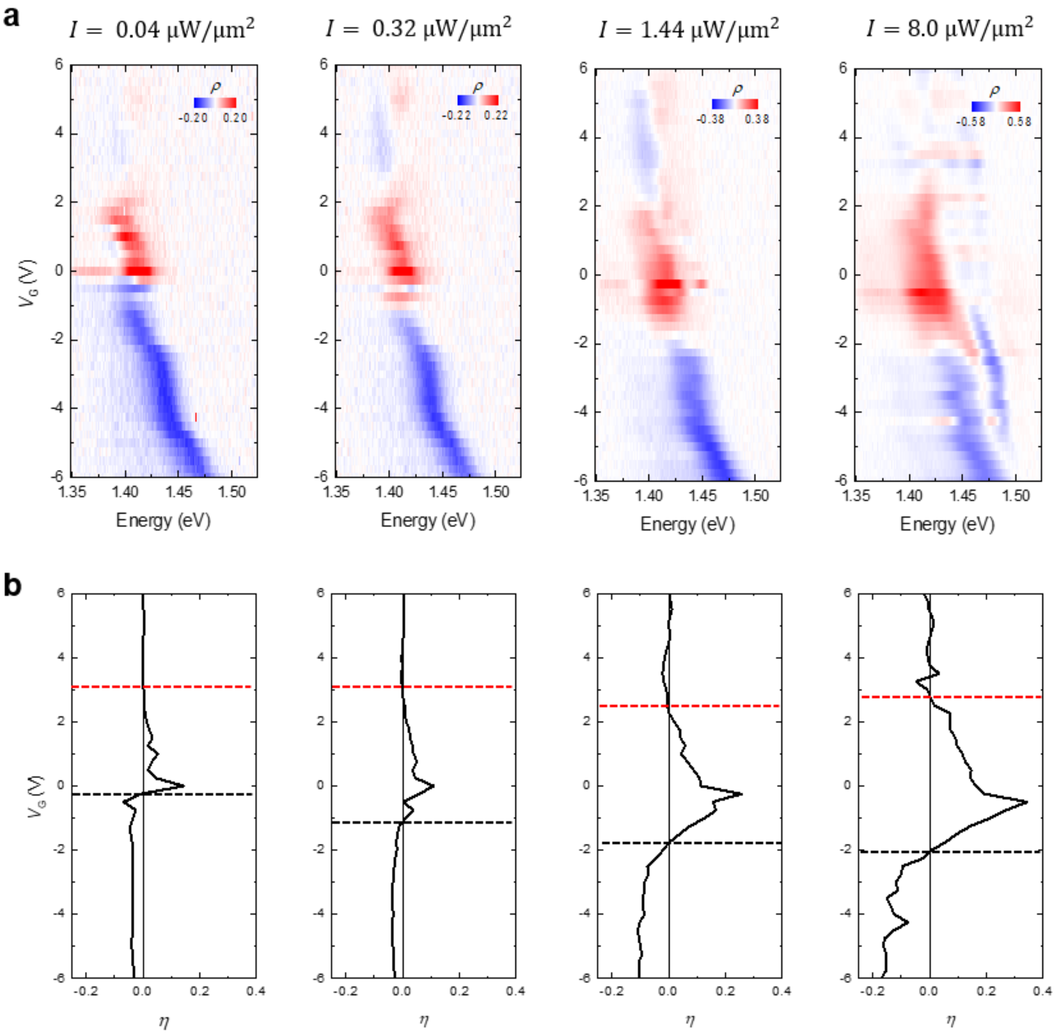

Supplementary Figure 4 | Gate-dependent degree of circular polarization and the valley polarization with increasing the optical excitation intensity. a Interlayer exciton degree of circular polarization ($\rho = \frac{\sigma^+/\sigma^+ - \sigma^+/\sigma^-}{\sigma^+/\sigma^+ + \sigma^+/\sigma^-}$) as a function of V_G at selected laser intensity I. **b** Corresponding valley polarization as a function of V_G at I. The valley polarization (η) is defined as $\eta = \frac{I_{\sigma^+/\sigma^+} - I_{\sigma^+/\sigma^-}}{I_{\sigma^+/\sigma^+} + I_{\sigma^+/\sigma^-}}$, where $I_{\sigma^+/\sigma^+(\sigma^-)}$ is the spectrally integrated PL intensity at the σ^+-$\sigma^+(\sigma^-)$ excitation-detection circular polarization configurations. Red (black) dashed-line indicates the n(p)-doping threshold voltage for each I.

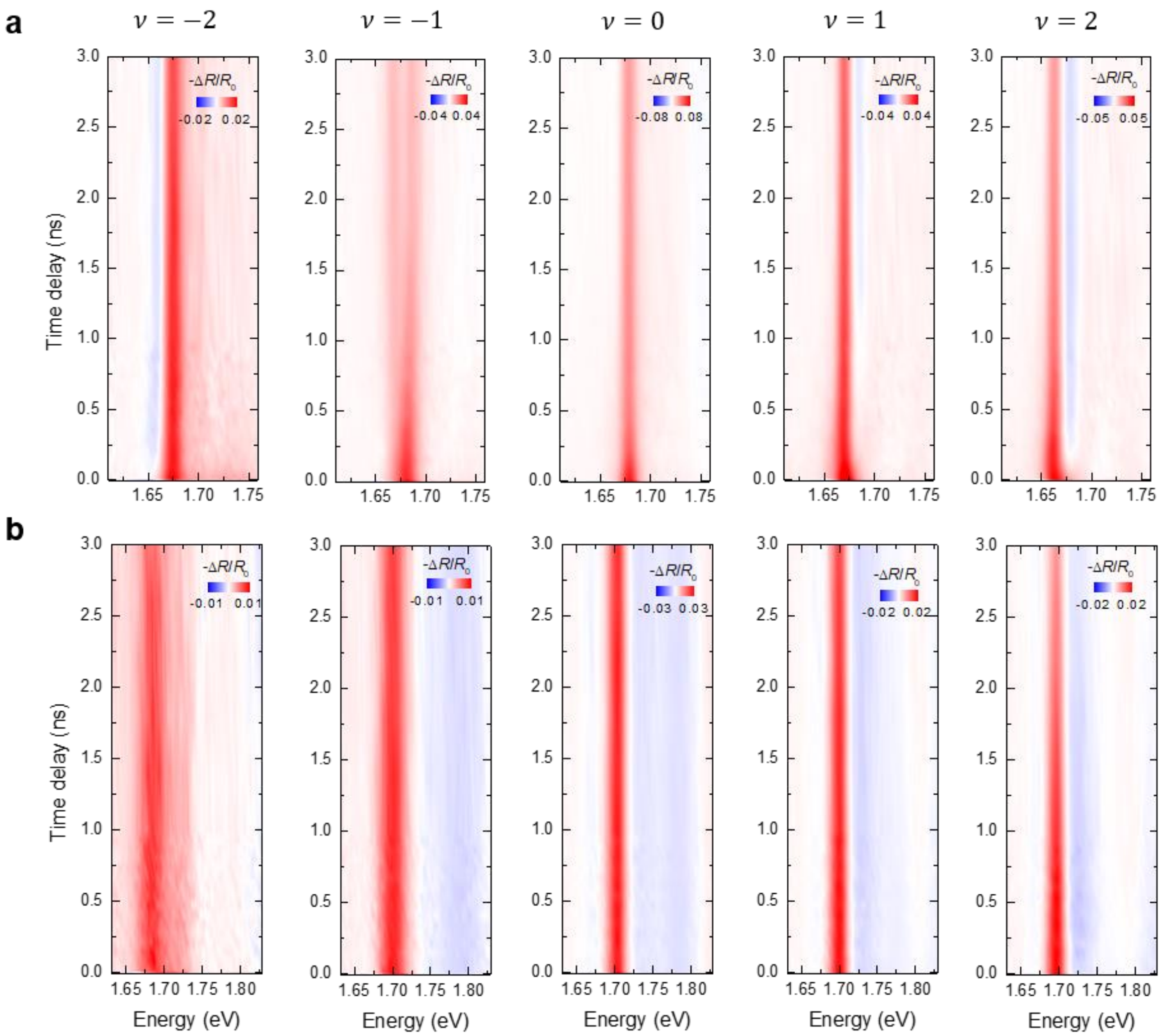

Supplementary Figure 5 | Time-resolved differential reflection changes at various integer fillings. a $\Delta R/R_0$ contour plots as a function of the probe photon energy and pump-probe delay at various integer fillings (from $\nu = -2$ to 2) on device R1. Spectral blueshifts are observed in the n-doped case. $\Delta R/R_0$ transients do not change significantly after $\Delta t = 0.1$ ns, which demonstrates the dynamics of the long-lived interlayer excitons. **b** The same plots for the device H1. The dynamics of the interlayer exciton is observed with modest blueshifts except at $\nu = -2$. The measurements are performed under the pump-photon energy of 2.0 eV with F of 12 $\mu J/cm^2$.

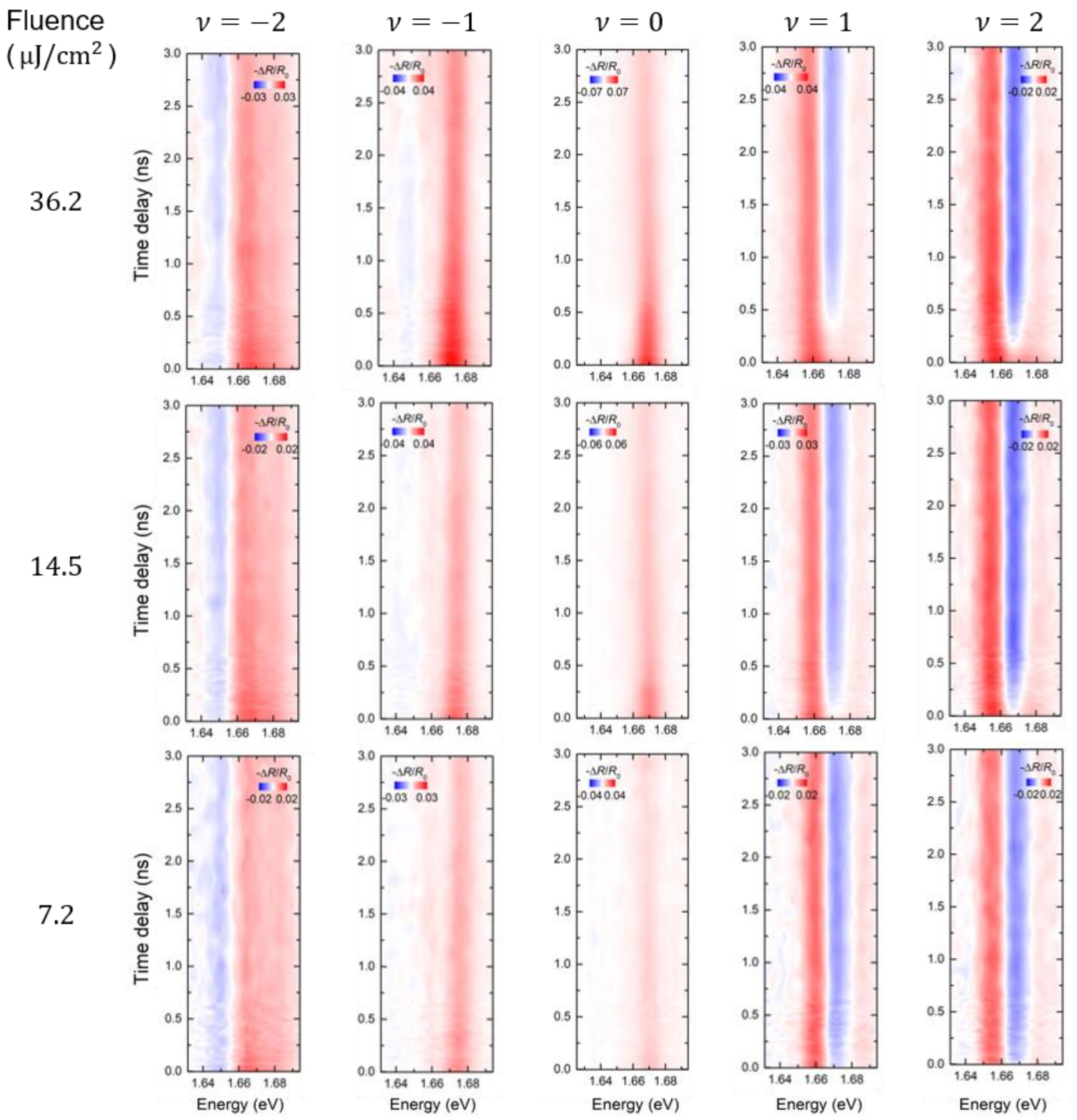

Supplementary Figure 6 | Fluence-dependent transient $\Delta R/R_0$ contour plots with various integer fillings. Full fluence-dependent contour plots with integer fillings ν from -2 to 2. The spectrally integrated signal reflects the population of photoexcited carriers. From the temporal evolution of this population, rise and decay time constants are extracted, which are summarized in Fig. 5 of the main text.

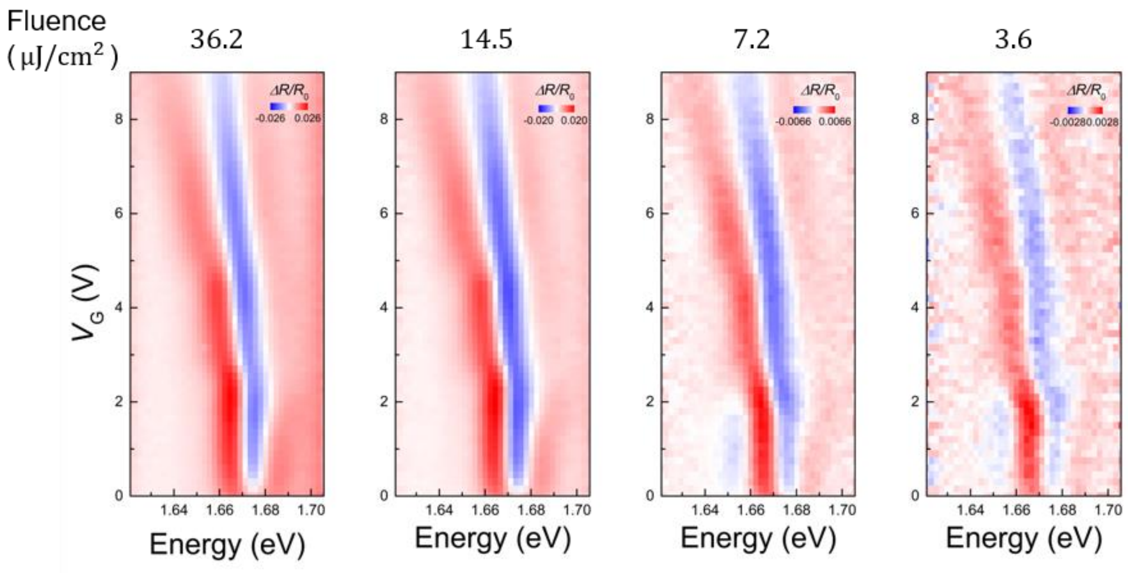

Supplementary Figure 7 | Fluence-dependent transient $\Delta R/R_0$ spectra at time delay 2 ns. The contour plots measured at $\Delta t = 2$ ns are shown as a function of probe photon energy and V_G to monitor the interlayer exciton site transition. The pump photon energy of 1.75 eV is employed which is lower than the one used in Fig. 4d. Despite the variation of the pump photon energy, an enhanced Pauli blocking signal is consistently observed in the n-doping regime across all F.

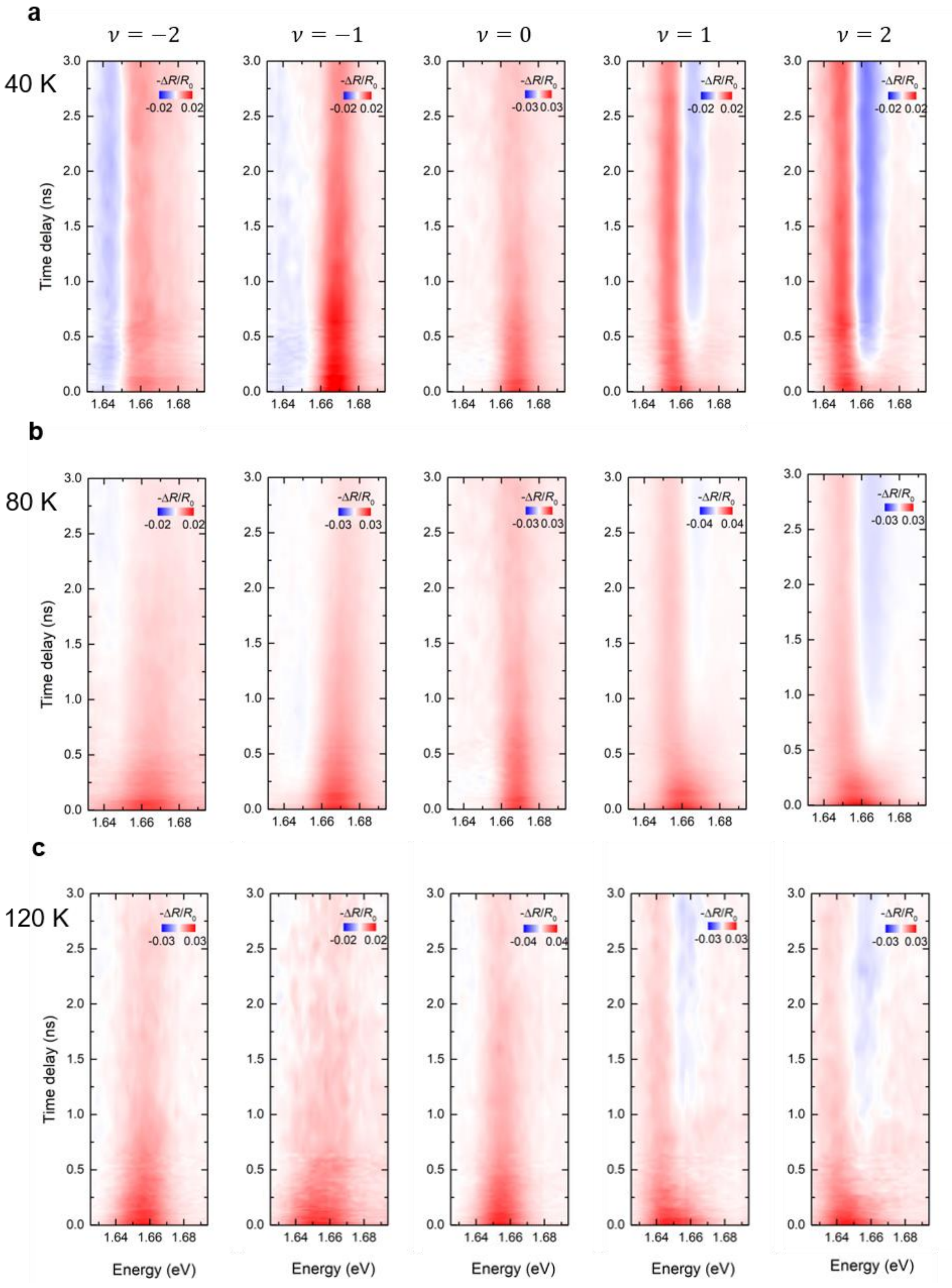
a
b
c
$\nu = -2$
$\nu = -1$
$\nu = 0$
$\nu = 1$
$\nu = 2$
40 K
80 K
120 K
Time delay (ns)
Energy (eV)
$-\Delta R/R_0$

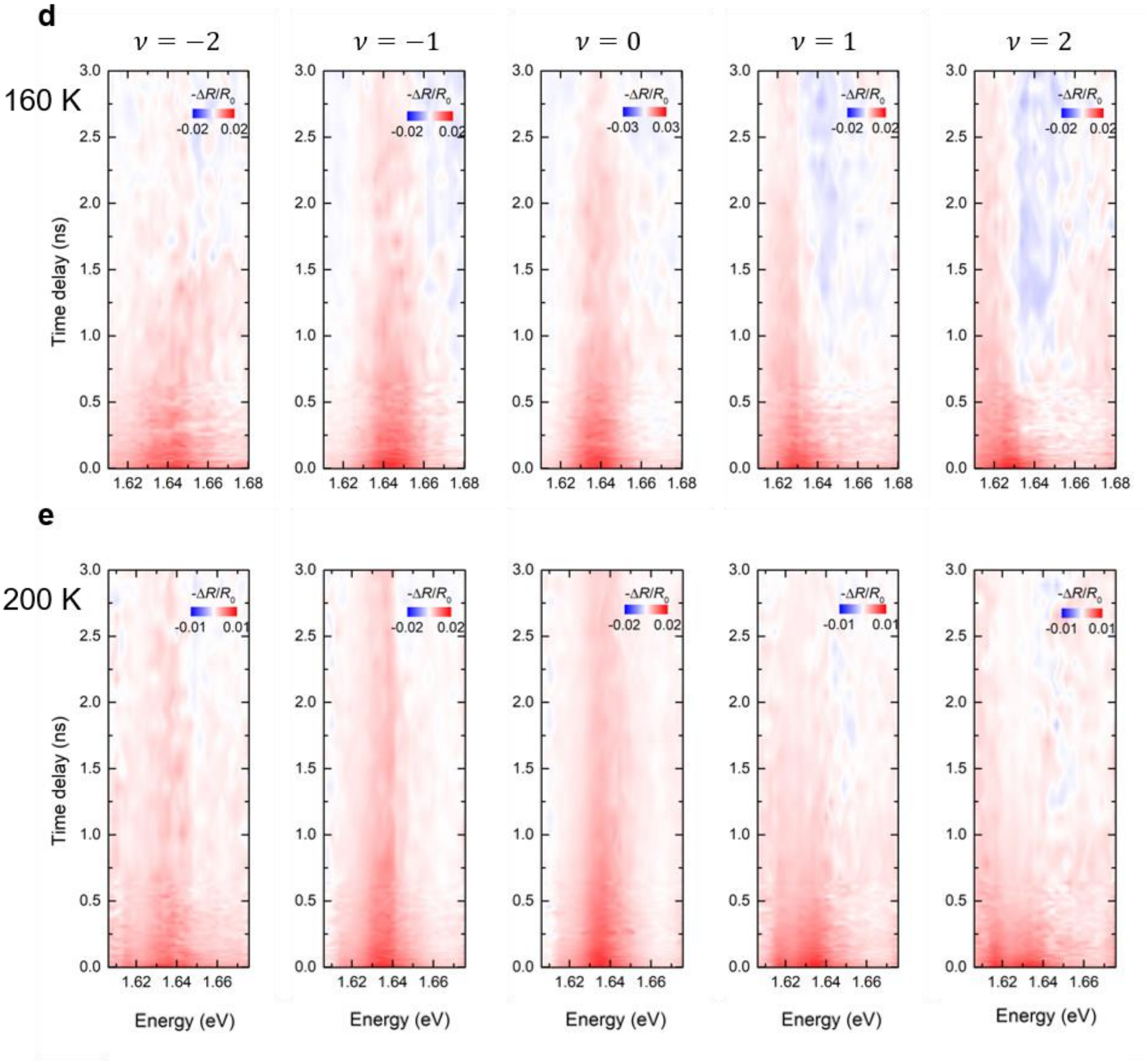

Supplementary Figure 8 | Temperature-dependent transient $\Delta R/R_0$ contour plots with various integer fillings. a-e, $\Delta R/R_0$ contour plots as a function of the probe photon energy and the time delay are illustrated at temperatures 40, 80, 120, 160, and 200 K, respectively. An enhanced blueshift signal at $\nu = 2$ is evident at 40 K, particularly after the long time delay (e.g. 2 ns). This finding is attributed to the correlation-driven exciton site transition, a topic elaborated in the main text. With increasing the temperature up to 200 K, the distinct blueshift signature gradually diminishes. When the temperature reaches 200 K, the ν-dependent spectral shift exhibits

marginal changes. Based on the fact that the thermal-activation temperature for Mott-Hubbard gap is around 150-180 K in WSe_2/WS_2[9], it suggests that the microscopic origin of the dynamics of exciton site transition is related to correlation effects.

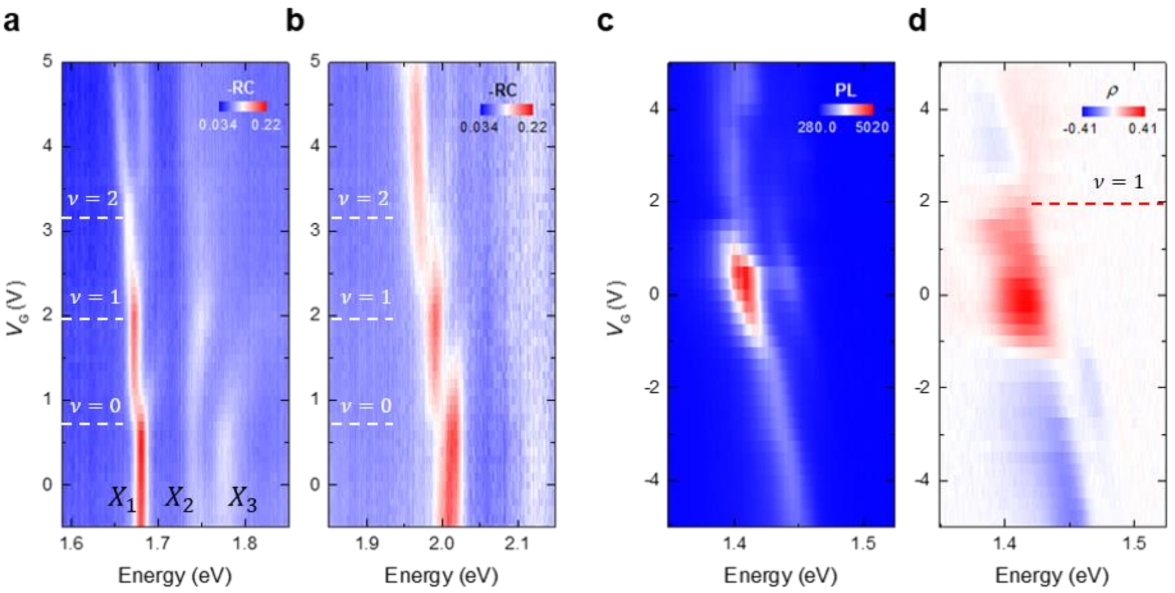

Supplementary Figure 9 | Reflection contrast and photoluminescence measurement on device R2. a,b Gate-dependent RC data on device R2 near the A exciton resonances in WSe_2 and WS_2, respectively. **a** Three distinct moiré intralayer excitons are resolved at $V_G = 0$ V; the data are essentially the same as R1 (Fig. 2a). **c,d** Gate-dependent PL and the corresponding degree of circular polarization (ρ), respectively. Optical excitation is 632.8 nm with intensity of 8.0 $\mu W/\mu m^2$. **d** Polarization switching occurs near the Mott insulating state ($V_G = 2$ V), which is consistent with the device R1.

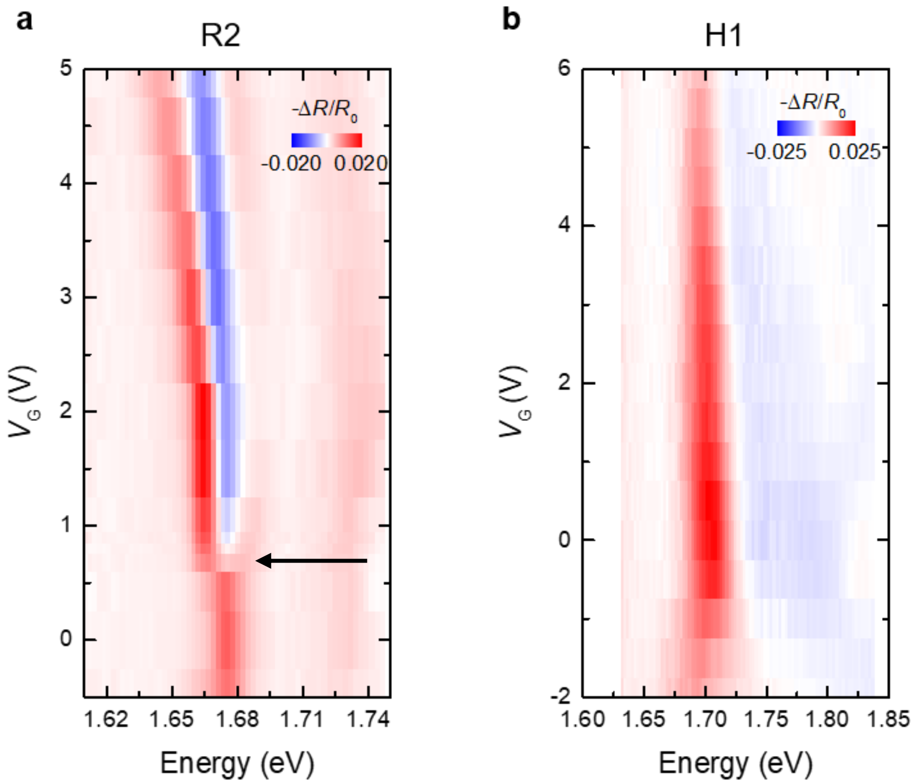

Supplementary Figure 10 | Comparison of gate-dependent pump-probe measurements on device R2 and device H1. a Gate-dependent transient absorption changes at a time delay of 2 ns for the device R2. Abrupt spectral blueshift (black arrow) appears upon n-doping which is consistent with Fig. 4d. Correlation-driven exciton site transition accompanied with polarization switching and the corresponding Pauli blocking are demonstrated in both R1 and R2. **b** Same plot for the device H1. The absence of abrupt spectral change within the entire gate voltage indicates that there is no exciton site transition. In addition, this observation is consistent with the absence of polarization switching, as illustrated in Fig. 2f.

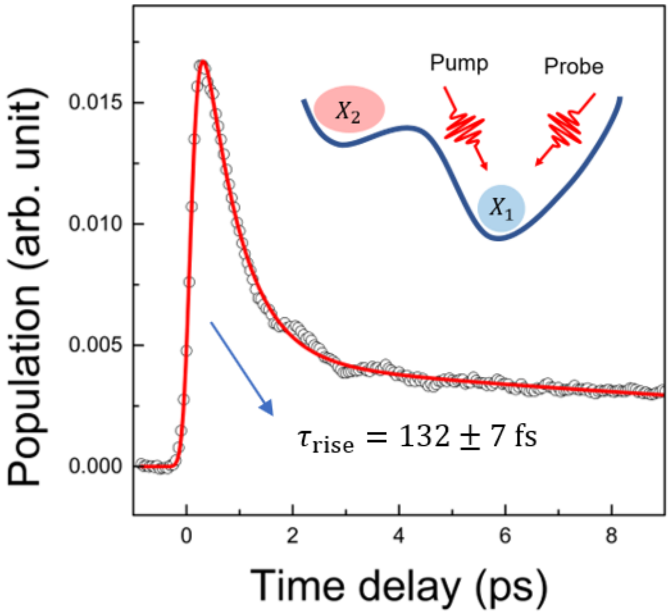

Supplementary Figure 11 | Degenerate pump-probe measurement at X_1 exciton (1.68 eV). Transient population dynamics is obtained by a spectral integration of $\Delta R/R_0$. We note that the fastest rise time constant (τ_{rise}) is obtained when the exciton is resonantly excited. In this case, τ_{rise} reaches a value of 132 ± 7 fs. We employ linearly cross-polarized pump and probe geometry to filter out the scattering of pump pulse.

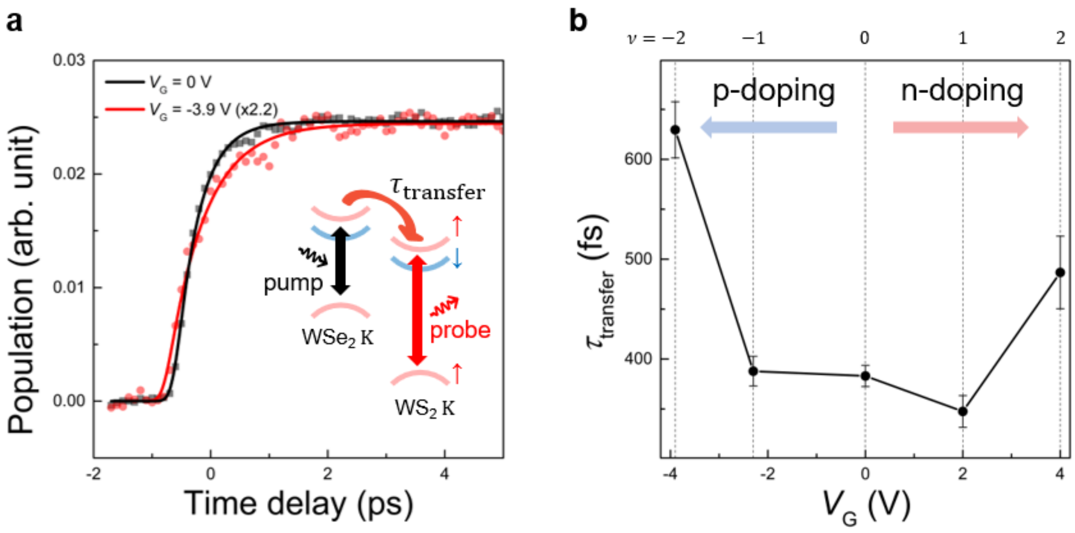

Supplementary Figure 12 | V_G -dependent charge transfer dynamics in WSe_2/WS_2 heterobilayers. a Temporal evolution of carrier population at the WS_2 A-exciton resonance when pump excitation takes place in the WSe_2/WS_2 heterobilayers. We extract the charge transfer time ($\tau_{transfer}$) by monitoring the rise dynamics of the population signals. $\tau_{transfer}$ represents the time for photoexcited electron separation via interfacial charge transfer from WSe_2 to WS_2 due to type-II band alignment (inset). While we estimate $\tau_{transfer} = 383 \pm 10$ fs at $V_G = 0$ V (black squares), $\tau_{transfer}$ becomes longer to 629 ± 28 fs at $V_G = -3.9$ V (red circles). Each solid line corresponds to the biexponential fits. **b** Measured V_G-dependent $\tau_{transfer}$. Note that $\tau_{transfer}$ is significantly long when the doping increases, see the cases of $V_G = -3.9$ V and $V_G = 4$ V. The error bars are obtained from the fits.

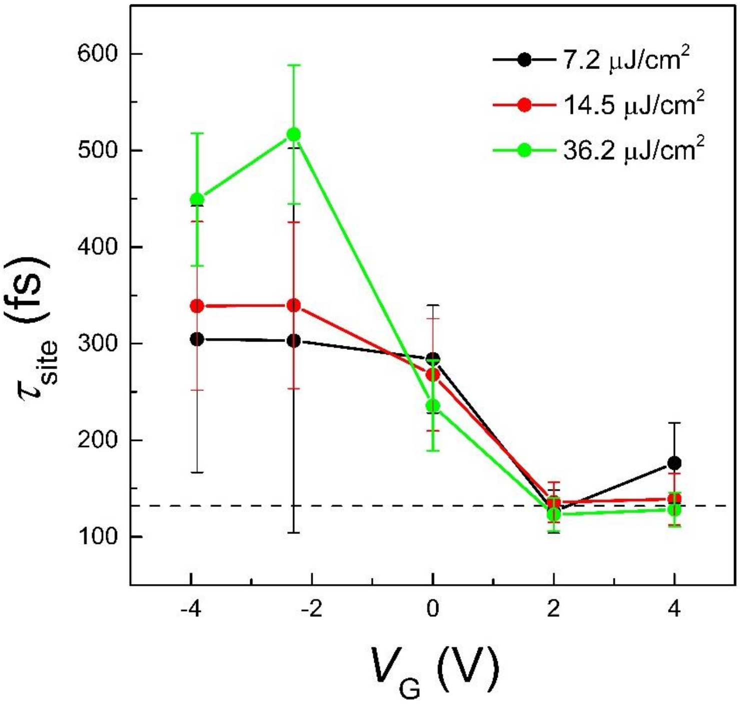

Supplementary Figure 13 | V_G-dependent τ_{site} for various F . The site transition time (τ_{site}) is measured at various F of 7.2 (black), 14.5 (red), 36.2 $\mu J/cm^2$ (green). Across all F, τ_{site} exhibits a decreased feature until it reaches the temporal resolution limit of 132 fs (dashed black line) for $V_G \geq 2$ V. Upon p-doping, τ_{site} not only shows an increased feature compared to the neutral regime, but also becomes longer with higher F. The error bars are obtained from the fits

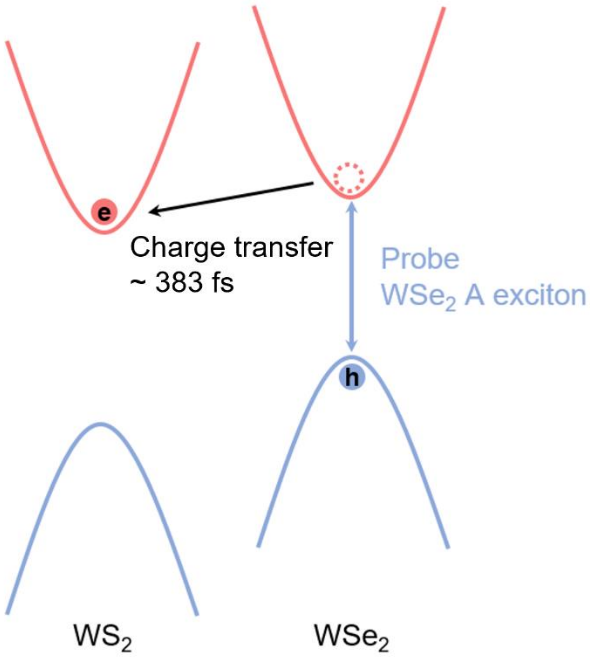

Supplementary Figure 14 | Schematic of the probing interlayer hole dynamics. The valence and conduction bands are depicted as solid blue and red lines, respectively. We examine interlayer hole dynamics through probing the A exciton of WSe_2, denoted by the blue arrow, while the black arrow indicates charge transfer.

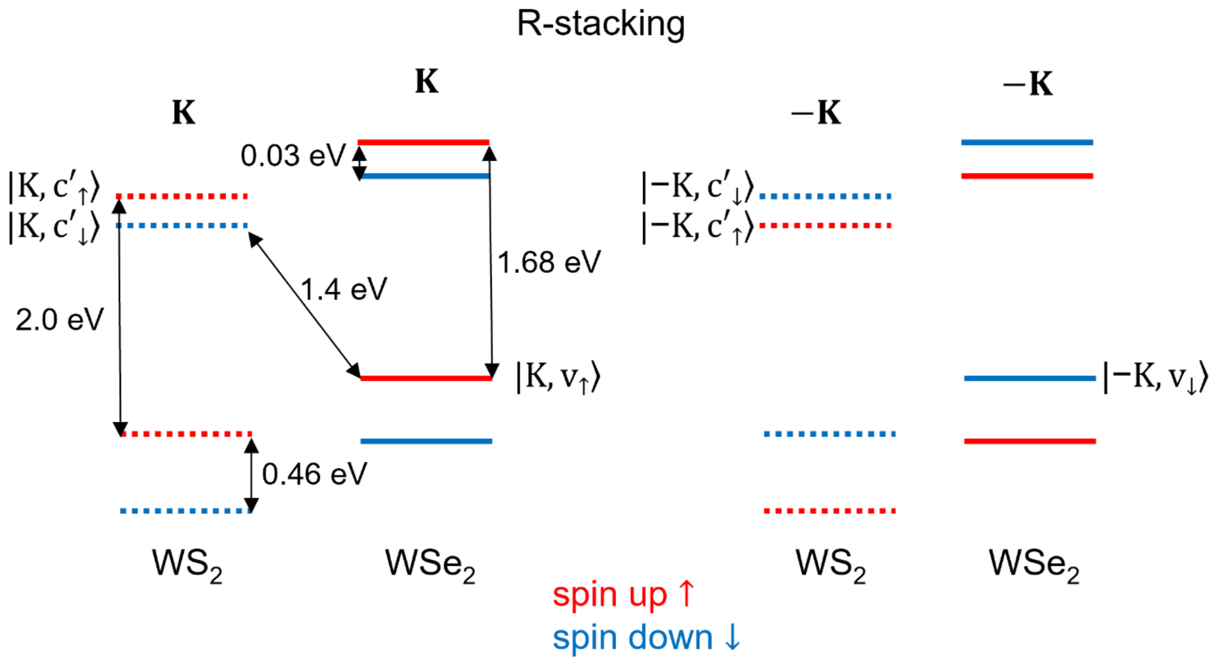

Supplementary Figure 15 | Band alignment in the R-stacked WSe_2/WS_2. Type II band alignment of R-stacked WSe_2/WS_2, where K(−K) is the ±K valley index, c′ (v) is conduction (valence) band of WS_2 (WSe_2) and the red (blue) band indicates the up-spin (down-spin). Due to R-stacked configuration, the K valley of WSe_2 and WS_2 are aligned in the momentum space. Corresponding optical selection rules for interlayer transitions are shown in Supplementary Table 1.

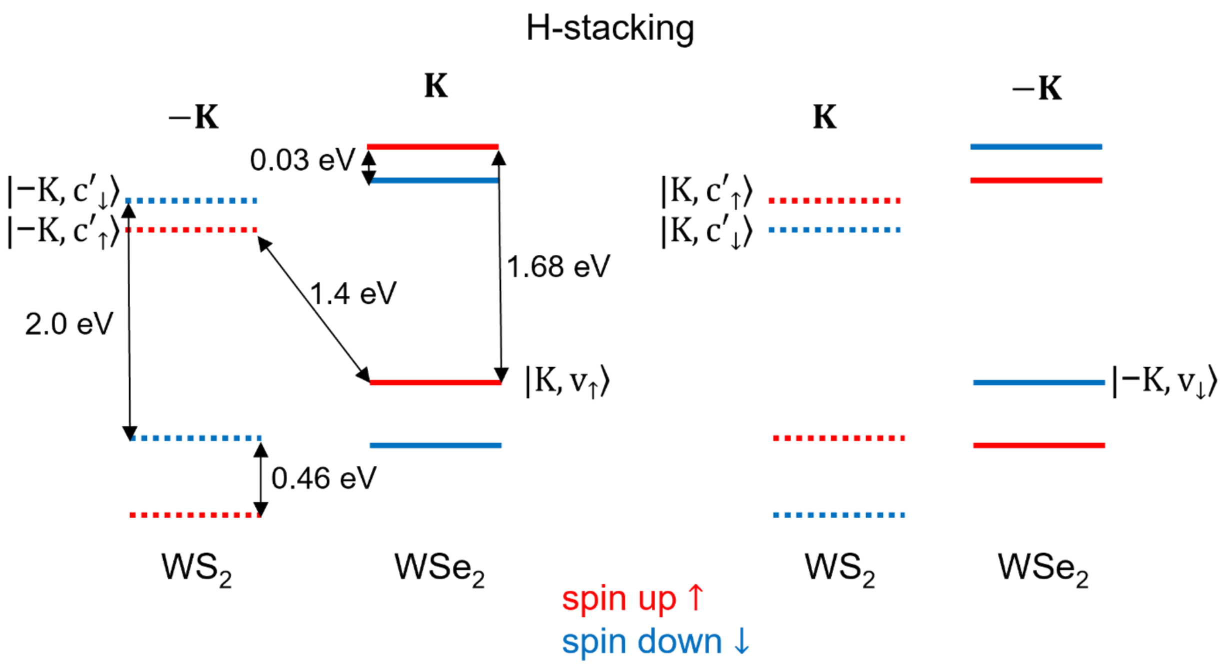

Supplementary Figure 16 | Band alignment in the H-stacked WSe_2/WS_2. Same plot as Supplementary Figure 15, but for the H-stacked one. Corresponding optical selection rules for interlayer transitions are shown in Supplementary Table 2.

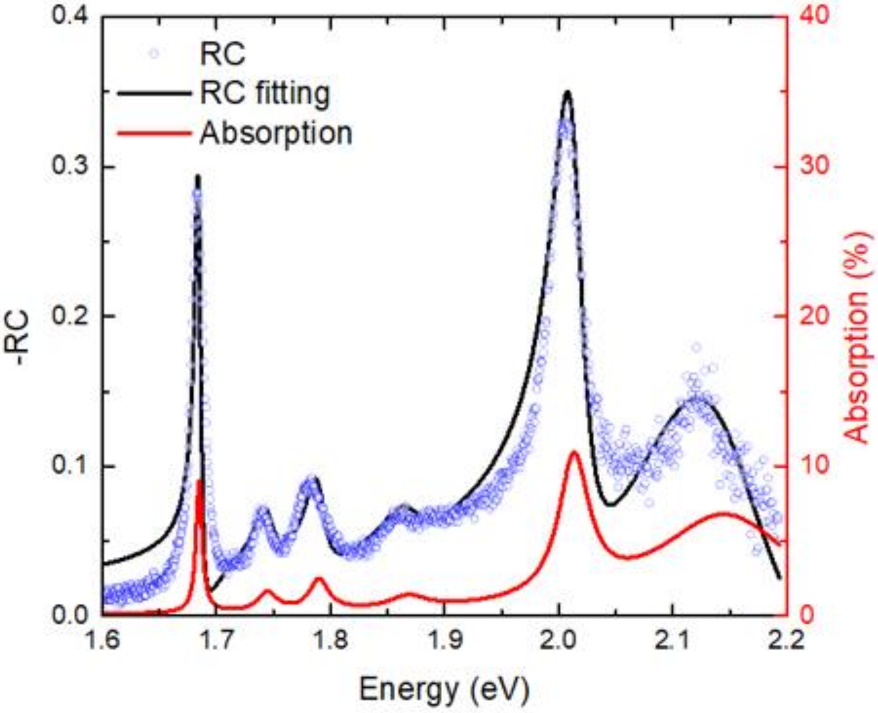

Supplementary Figure 17 | Extraction of absorption by the transfer-matrix method. Reflection contrast spectrum at 4 K (blue open circles) and the corresponding fit (black line) by using the transfer-matrix method. Corresponding absorption can be extracted which is plotted as red line (right axis) by the summation of the independent Lorentz oscillators.

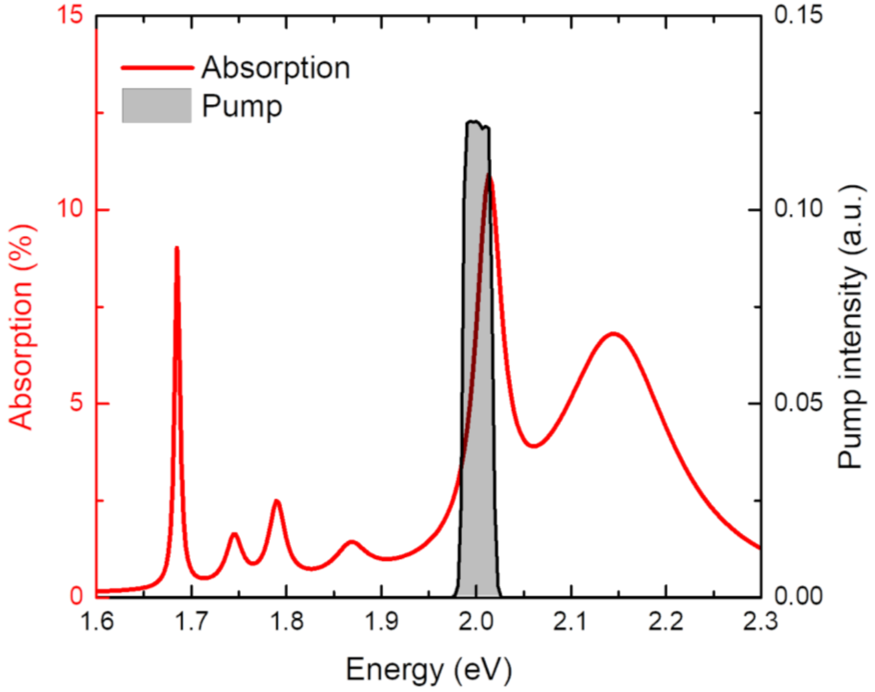

Supplementary Figure 18 | Estimation of total absorption (A_{tot}) and photoexcited exciton density (n_{ex}). Extracted absorption and pump intensity are plotted as a red (left axis) and black line (right axis), respectively. Calculated total absorption is approximately 0.0748 and corresponding photoexcited exciton density (n_{ex}) is $2.79 \times 10^{12}\ \mathrm{cm}^{-2}$ for the pump fluence of $12\ \mu\mathrm{J/cm}^2$.

References

1 Ceballos, F., Bellus, M. Z., Chiu, H.-Y. & Zhao, H. Ultrafast charge separation and indirect exciton formation in a MoS_2–$MoSe_2$ van der Waals heterostructure. *ACS Nano* **8**, 12717-12724 (2014).

2 Hong, X. et al. Ultrafast charge transfer in atomically thin MoS_2/WS_2 heterostructures. *Nat. Nanotechnol.* **9**, 682-686 (2014).

3 Jin, C. et al. Imaging of pure spin-valley diffusion current in WS_2-WSe_2 heterostructures. *Science* **360**, 893-896 (2018).

4 Yuan, L. et al. Twist-angle-dependent interlayer exciton diffusion in WS_2-WSe_2 heterobilayers. *Nat. Mater.* **19**, 617-623 (2020).

5 Naik, M. H. et al. Intralayer charge-transfer moiré excitons in van der Waals superlattices. *Nature* **609**, 52-57 (2022).

6 Wang, X. et al. Intercell moiré exciton complexes in electron lattices. *Nat. Mater.* **22**, 599-604 (2023).

7 Wu, F., Lovorn, T., Tutuc, E. & MacDonald, A. H. Hubbard model physics in transition metal dichalcogenide moiré bands. *Phys. Rev. Lett.* **121**, 026402 (2018).

8 Yang, J. et al. Spectroscopy signatures of electron correlations in a trilayer graphene/hBN moiré superlattice. *Science* **375**, 1295-1299 (2022).

9 Tang, Y. et al. Simulation of Hubbard model physics in WSe_2/WS_2 moiré superlattices. *Nature* **579**, 353-358 (2020).

10 Xiong, R. et al. Correlated insulator of excitons in WSe_2/WS_2 moiré superlattices. *Science* **380**, 860-864 (2023).

11 Regan, E. C. et al. Mott and generalized Wigner crystal states in WSe_2/WS_2 moiré superlattices. *Nature* **579**, 359-363 (2020).

12 Zhang, Y., Yuan, N. F. Q. & Fu, L. Moiré quantum chemistry: Charge transfer in transition metal dichalcogenide superlattices. *Phys. Rev. B* **102** (2020).

13 Katsidis, C. C. & Siapkas, D. I. General transfer-matrix method for optical multilayer systems with coherent, partially coherent, and incoherent interference. *Appl. Opt.* **41**, 3978-3987 (2002).

14 Zhan, T., Shi, X., Dai, Y., Liu, X. & Zi, J. Transfer matrix method for optics in graphene layers. *J. Phys. Condens. Matter* **25**, 215301 (2013).

15 Robert, C. et al. Optical spectroscopy of excited exciton states in MoS_2 monolayers in van der Waals heterostructures. *Phys. Rev. Mater.* **2** (2018).

16 Yoon, Y. et al. Charge Transfer Dynamics in $MoSe_2$/hBN/WSe_2 Heterostructures. *Nano Lett.* **22**, 10140-10146 (2022).

17 Prasankumar, R. P. & Taylor, A. J. *Optical techniques for solid-state materials characterization*. (CRC press, 2016).

18 Yu, H., Liu, G.-B. & Yao, W. Brightened spin-triplet interlayer excitons and optical selection rules in van der Waals heterobilayers. *2D Mater.* **5** (2018).

19 Yu, H., Liu, G.-B., Tang, J., Xu, X. & Yao, W. Moiré excitons: From programmable quantum emitter arrays to spin-orbit–coupled artificial lattices. *Sci. Adv.* **3**, e1701696 (2017).

20 Jin, C. et al. Identification of spin, valley and moiré quasi-angular momentum of interlayer excitons. *Nat. Phys.* **15**, 1140-1144 (2019).

21 Ciarrocchi, A. et al. Polarization switching and electrical control of interlayer excitons in two-dimensional van der Waals heterostructures. *Nat. Photon.* **13**, 131-136 (2019).
